# Hepatic disease control in patients with intrahepatic cholangiocarcinoma correlates with overall survival

**DOI:** 10.1002/cam4.5925

**Published:** 2023-04-16

**Authors:** Kevin C. Soares, Joshua S. Jolissaint, Sarah M. McIntyre, Kenneth P. Seier, Mithat Gönen, Carlie Sigel, Naaz Nasar, Andrea Cercek, James J. Harding, Nancy E. Kemeny, Louise C. Connell, Bas Groot Koerkamp, Vinod P. Balachandran, Michael I. D'Angelica, Jeffrey A. Drebin, T. Peter Kingham, Alice C. Wei, William R. Jarnagin

**Affiliations:** ^1^ Department of Surgery, Hepatopancreatobiliary Service Memorial Sloan Kettering Cancer Center New York New York USA; ^2^ Department of Surgery Brigham and Women's Hospital Boston Massachusetts USA; ^3^ Department of Epidemiology and Biostatistics Memorial Sloan Kettering Cancer Center New York New York USA; ^4^ Department of Pathology Memorial Sloan Kettering Cancer Center New York New York USA; ^5^ Department of Medicine Memorial Sloan Kettering Cancer Center New York New York USA; ^6^ Department of Surgery Erasmus Medical Center Rotterdam The Netherlands

**Keywords:** bile duct cancer, biliary neoplasm, cholangiocarcinoma, hepatic artery pump, locoregional therapy, regional chemotherapy

## Abstract

**Purpose:**

The role of locoregional therapy compared to systemic chemotherapy (SYS) for unresectable intrahepatic cholangiocarcinoma (IHC) remains controversial. The importance of hepatic disease control, either as initial or salvage therapy, is also unclear. We compared overall survival (OS) in patients treated with resection, hepatic arterial infusion pump (HAIP) chemotherapy, or SYS as it relates to hepatic recurrence or progression. We also evaluated recurrence after resection to determine the efficacy of locoregional salvage therapy.

**Patients and Methods:**

In this single‐institution retrospective analysis, patients with biopsy‐proven IHC treated with either curative‐intent resection, HAIP (with or without SYS), or SYS alone were analyzed. Propensity score matching (PSM) was used to compare patients with liver‐limited, advanced disease treated with HAIP versus SYS. The impact of locoregional salvage therapies in patients with liver‐limited recurrence was analyzed in the resection cohort.

**Results:**

From 2000 to 2017, 714 patients with IHC were treated, 219 (30.7%) with resectable disease, 316 (44.3%) with locally advanced disease, and 179 (25.1%) with metastatic disease. Resected patients were less likely to recur or progress in the liver (hazard ratio [HR] 0.41, 95% CI 0.34–0.45) versus those that received HAIP or SYS (HR 0.58, 95% CI 0.50–0.65 vs. HR 0.63, 95% CI 0.57–0.69, respectively). In resected patients, 161 (64.4%) recurred, with 65 liver‐only recurrences. Thirty of these patients received subsequent locoregional therapy. On multivariable analysis, locoregional therapy was associated with improved OS after isolated liver recurrence (HR 0.46, 95% CI 0.29–0.75; *p* = 0.002). In patients with locally advanced unresectable or multifocal liver disease (with or without distant organ metastases), PSM demonstrated improved hepatic progression‐free survival in patients treated with HAIP versus SYS (HR 0.65; 95% CI 0.46–0.91; *p* = 0.01), which correlated with improved OS (HR 0.59, 95% CI 0.43–0.80; *p* < 0.001).

**Conclusion:**

In patients with liver‐limited IHC, hepatic disease control is associated with improved OS, emphasizing the potential importance of liver‐directed therapy.

## 1 INTRODUCTION

Intrahepatic cholangiocarcinoma (IHC) comprises approximately 10% of all liver cancer cases, and its incidence has steadily increased over time.^1^, ^2^, ^3^ Most patients present with locally advanced or metastatic disease at diagnosis and are treated with systemic chemotherapy (SYS) alone. Unfortunately, standard cytotoxic regimens have limited effectiveness, and while recent studies have demonstrated encouraging results with targeted therapies, these agents are only effective in a small subset of patients with specific genomic alterations.^4^, ^5^, ^6^, ^7^ Resection offers the best potential for long‐term disease control but is only possible in one‐third of patients, and ultimately >60% of resected patients will recur.^8^, ^9^

Failure to achieve hepatic disease control, due to either recurrence after resection or progression while on treatment for advanced disease, is common in IHC. As a result, liver‐directed therapies may be appropriate and potentially effective in a large subgroup of patients.^10^ In a recently completed Phase II study of hepatic artery infusion pump (HAIP) floxuridine (FUDR) combined with systemic gemcitabine and oxaliplatin (GEM/OX) in patients with unresectable IHC (*N* = 38), we reported a median overall survival (OS) of 25.0 months despite a 47% incidence of lymph node metastasis.^11^ Conversely, the 3‐year OS of liver‐only disease in patients treated with gemcitabine and cisplatin alone is 3%.^12^ However, the effectiveness of locoregional therapy when compared to SYS alone in patients with advanced disease has yet to be compared in a randomized clinical trial and remains controversial. Likewise, the role of locoregional treatment on intrahepatic recurrence following resection has not been established.

The goal of this study was to investigate the impact of hepatic disease control in IHC and its implications for OS. We analyzed a large, single‐institution series of patients with biopsy‐proven IHC treated by resection, HAIP FUDR, and SYS alone. We hypothesized that control of hepatic disease would be associated with improved survival, both in patients with liver‐limited resectable and unresectable disease (without distant organ metastasis). Additionally, we analyzed a separate cohort of patients with liver‐limited recurrence after curative resection to evaluate the impact of liver‐directed locoregional therapy as salvage therapy in this patient population.

## 2 METHODS

### 2.1 Patients

This was a retrospective analysis of a prospectively maintained database of patients with biopsy‐proven IHC from a single institution treated between 2000 and 2017 with either curative‐intent resection, HAIP (with or without SYS), or SYS alone. Approval from the Institutional Review Board at Memorial Sloan Kettering Cancer Center (MSKCC) was obtained prior to data collection (protocol #16‐698).

The operative approach and outcomes in patients with resectable IHC have been previously described.^13^, ^14^ The technical details of HAIP placement and outcomes from this therapy have similarly been described.^11^, ^15^, ^16^, ^17^ A portal lymphadenectomy or targeted excisional lymphadenectomy in the porta hepatitis was performed in the HAIP and resected cohorts at the surgeon's discretion. Multifocal hepatic disease was defined as the presence of intrahepatic metastases or satellite nodules, either radiographically, intraoperatively, or on final pathology. Locally advanced unresectable disease was defined as anatomically unresectable disease due to insufficient future liver remnant or inability to obtain a margin negative resection. Tumor size was defined as the largest diameter of the tumor in the pathology specimen for resected patients, or the largest diameter seen on imaging for patients who were treated with HAIP. Patients who received HAIP but ultimately underwent curative‐intent resection were analyzed in the HAIP cohort on an intention‐to‐treat basis. The impact of locoregional salvage therapies in patients with liver‐limited recurrence was analyzed in the resection cohort.

### 2.2 Statistical analysis

Continuous variables are described with median and range and compared using Wilcoxon rank sum test. Categorical variables are described with count and percentage and compared using Fischer's exact test. OS was defined as time from initial treatment initiation (resection, HAIP placement, or initiation of SYS) to the date of death or censored at the date of last follow‐up. Progression‐free survival (PFS) was measured from initial treatment initiation to time of first progression. A competing risk framework was used to analyze PFS outcomes in which death without progression was treated as a competing risk. Site‐specific PFS treated recurrence at a different site as a competing risk. PFS outcomes were visualized using cumulative incidence function (CIF) curves and compared using Fine and Gray test. OS was visualized using the Kaplan–Meier method, and the log‐rank test was used to assess differences between treatment groups.

Propensity score matching (PSM) was used to assess HAIP versus SYS. A propensity score model was built using logistic regression and included age, sex, race, lymphadenopathy, multifocality, and tumor grade. Patients were matched by propensity scores using a 1:1 greedy matching algorithm with a 0.25 (04.16%) standard deviation caliper. Balance of factors used in the PSM was assessed using Wilcoxon sign rank test for continuous variables, McNemar's test for categorical variables with two levels, and GEE model for categorical variables with >2 levels to account for correlation between matched pairs. Time to event outcomes were compared between matched groups using Cox proportional hazards models for OS and Fine and Gray models for PFS outcomes, with a robust sandwich estimator to account for matching.

Within the resected cohort, Cox proportional hazard models with time‐dependent covariates were used to estimate the effect of recurrence on OS. An exploratory subgroup analysis on resected patients that recurred was performed to evaluate locoregional salvage therapy in patients with liver‐limited recurrence. *p* < 0.05 was considered statistically significant. Data were analyzed using SAS Version 9.4 (SAS Institute) and R 4.0.2 (R Foundation for Statistical Computing).

## 3 RESULTS

**FIGURE 1:**
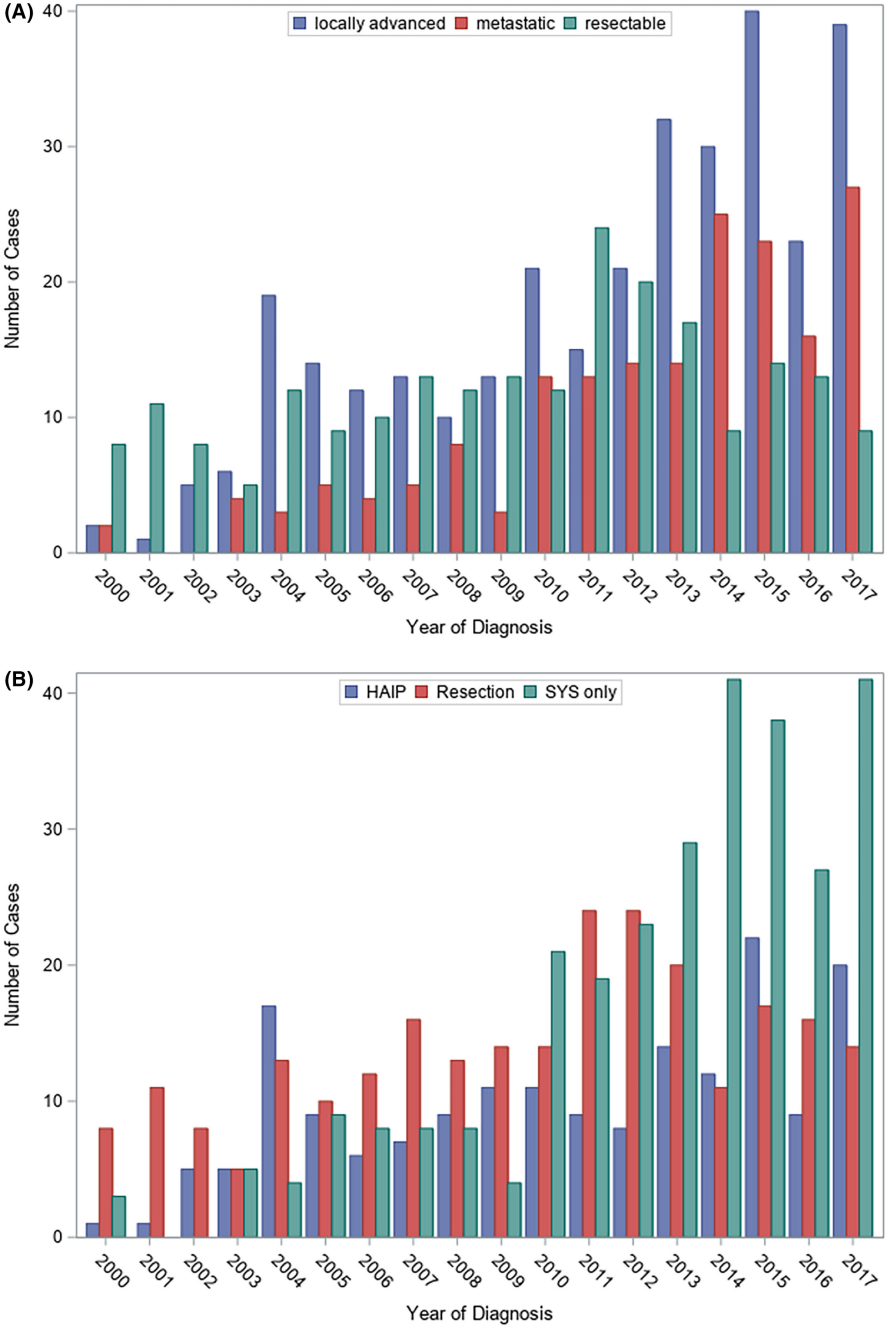
Intrahepatic cholangiocarcinoma patients (*n* = 714) by year of diagnosis stratified by (A) clinical presentation (resectable, locally advanced, or metastatic) and (B) treatment (resection, hepatic artery infusion pump [HAIP], or systemic chemotherapy alone [SYS]).

From 2000 to 2017, 714 consecutive patients were treated at MSKCC for IHC (Table 1). Of these, 219 patients (30.7%) presented with resectable disease, 316 (44.3%) presented with locally advanced, and 179 (25.1%) presented with metastatic disease (Figure 1A). It is our practice to offer resection or HAIP placement to patients with either no evidence of cirrhosis, or well‐compensated Child's A cirrhosis without portal hypertension. Similarly, patients treated with SYS were required to have adequate liver function. In our cohort, only 32/288 (11.1%) of patients treated with SYS alone had underlying cirrhosis. Patients with advanced underlying liver parenchymal disease, portal hypertension, or decompensated cirrhosis were typically not candidates for aggressive tumor‐directed therapy of any time. The percentage of patients presenting with unresectable disease and treated with SYS alone has increased in recent years, notably when splitting the cohort between 2011–2017 and 2000–2010 (49.6% vs. 25.4%, *p* < 0.001) (Figure 1B). The median follow‐up for the cohort was 31.8 months. In the overall cohort, 150 (21%) patients presented with extrahepatic disease, of whom 145 (96.7%) were treated with SYS alone. Gemcitabine plus cisplatin was the most common first‐line systemic regimen (*n* = 127, 44.1%) in SYS‐only patients. HAIP patients received either FUDR alone (*n* = 53, 30.1%) or FUDR combined with either gemcitabine (*n* = 27, 15.3%), irinotecan (*n* = 37, 21.0%), or gemcitabine plus oxaliplatin (*n* = 46, 26.1%).

**TABLE 1:** Patient characteristics.

	Total (*n* = 714)	HAIP (*n* = 176)	Resection (*n* = 250)	SYS (*n* = 288)	*p*‐Value
Age (years), median (range)	64.8 (19.0–92.3)	62.0 (30.1–85.7)	67.1 (19.0–88.6)	63.8 (27.6–92.3)	<0.001
Sex					0.069
Female	385 (53.9)	106 (60.2)	137 (54.8)	142 (49.3)	
Male	329 (46.1)	70 (39.8)	113 (45.2)	146 (50.7)	
Race					0.239
Other	91 (13.2)	17 (9.9)	31 (12.7)	43 (15.6)	
White	600 (86.8)	154 (90.1)	213 (87.3)	233 (84.4)	
Unknown	23	5	6	12	
Era					<0.001
2000–2010	276 (38.7)	82 (46.6)	124 (49.6)	70 (24.3)	
2011–2017	438 (61.3)	94 (53.4)	126 (50.4)	218 (75.7)	
Suspicious lymphadenopathy					<0.00
None	340 (47.7)	71 (40.3)	161 (64.7)	108 (37.5)	
Regional	187 (26.2)	46 (26.1)	46 (18.5)	95 (33)	
Distant	59 (8.3)	10 (5.7)	16 (6.4)	33 (11.5)	
Both	127 (17.8)	49 (27.8)	26 (10.4)	52 (18.1)	
Unknown	1	0	1	0	
Regional lymph nodes					<0.001
Negative	174 (24.4)	70 (39.8)	104 (41.6)	0 (0)	
pXn	446 (62.5)	55 (31.3)	103 (41.2)	288 (100)	
Positive	94 (13.2)	51 (29)	43 (17.2)	0 (0)	
Histologic grade					0.014
Well differentiated	15 (2.4)	6 (3.8)	3 (1.2)	6 (2.6)	
Moderately differentiated	395 (61.9)	88 (55.3)	171 (69.8)	136 (58.1)	
Poorly differentiated	228 (35.7)	65 (40.9)	71 (29)	92 (39.3)	
Unknown	76	17	5	54	
Multifocal liver disease					<0.001
No	304 (42.6)	47 (26.7)	185 (74)	72 (25)	
Yes	410 (57.4)	129 (73.3)	65 (26)	216 (75)	
Intra and extrahepatic disease	150 (21.0)	4 (2.3)	1 (0.4)	145 (50.3)	
Sites of extrahepatic disease					
Lung	61 (8.5)	2 (1.1)	0 (0)	59 (20.5)	
Bone	45 (6.3)	2 (1.1)	1 (0.4)	42 (14.6)	
Peritoneal	75 (10.5)	1 (0.6)	0 (0)	74 (25.7)	
Adrenal	11 (1.5)	0 (0)	0 (0)	11 (3.8)	
First‐line systemic chemotherapy					
Gemcitabine alone	34 (4.7)	1 (0.6)	0 (0)	33 (11.4)	
Gemcitabine/cisplatin	164 (22.9)	28 (15.9)	9 (3.6)	127 (44.1)	
Gemcitabine/oxaliplatin	59 (8.3)	10 (5.7)	2 (0.8)	47 (16.3)	
Gemcitabine/capecitabine	11 (1.5)	2 (1.1)	3 (1.2)	6 (2.1)	
Gemcitabine/ irinotecan	5 (0.7)	0 (0)	0 (0)	5 (1.7)	
FOLFOX/XELOX	18 (2.5)	2 (1.1)	0 (0)	16 (5.5)	
FOLFIRI/XELIRI	2 (0.3)	1 (0.6)	0 (0)	1 (0.3)	
Fluorouracil or capecitabine monotherapy	11 (1.5)	0 (0)	1 (0.4)	10 (3.5)	
Other	53 (7.4)	11 (6.3)	1 (0.4)	41 (14.2)	
≥2 lines of chemotherapy	174 (24.4)	15^a^ (8.5)	4 (1.6)	155 (53.8)	
HAIP chemotherapy regimen^b^					
FUDR alone	55 (7.7)	53 (30.1)	2 (0.8)		
FUDR + irinotecan	39 (5.5)	37 (21.0)	2 (0.8)		
FUDR + gemcitabine/oxaliplatin	51 (7.1)	46 (26.1)	5 (2)		
FUDR + gemcitabine	29 (4.1)	27 (15.3)	2 (0.8)		
FUDR + avastin	11 (1.5)	9 (5.1)	2 (0.8)		
FUDR + other agents	4 (0.5)	3 (1.7)	1 (0.4)		

*Note*: Data are *n* (%) unless noted.

Abbreviations: FOLFOX, fluorouracil, leucovorin, and oxaliplatin; FUDR, floxuridine; HAIP, hepatic arterial infusion pump; SYS, systemic chemotherapy only.

^a^ Prior to receiving HAIP.

^b^ One patient was never able to initiate HAIP chemotherapy.

As expected, resection was associated with an improved median OS (48.1 months, 95% CI 40.0–60.0) compared to HAIP (21.7 months, 95% CI 18.4–26.6) and SYS alone (10.8 months, 95% CI 8.9–12.9) (*p* < 0.001) (Figure S1A). Of particular note, liver resection and HAIP were associated with better liver disease control. Resected patients were significantly less likely to recur/progress in the liver compared to HAIP and SYS patients (2‐year CIF: 0.41, 95% CI 0.34–0.47 vs. 0.58, 95% CI 0.50–0.65 vs. 0.63, 95% CI 0.57–0.69, respectively, *p* < 0.001) (Figure S1B). While controlling for multifocal liver disease, tumor size, grade, and node status, a liver related recurrence was associated with a 7.9 (HR = 7.9, 95% CI 4.9–12.9; *p* < 0.001) fold increase in the risk of death and an extra hepatic recurrence was associated with a 9.6 (HR = 9.6, 95% CI 5.4–16.9; *p* < 0.001) fold increase in the risk of death.

**FIGURE 2:**
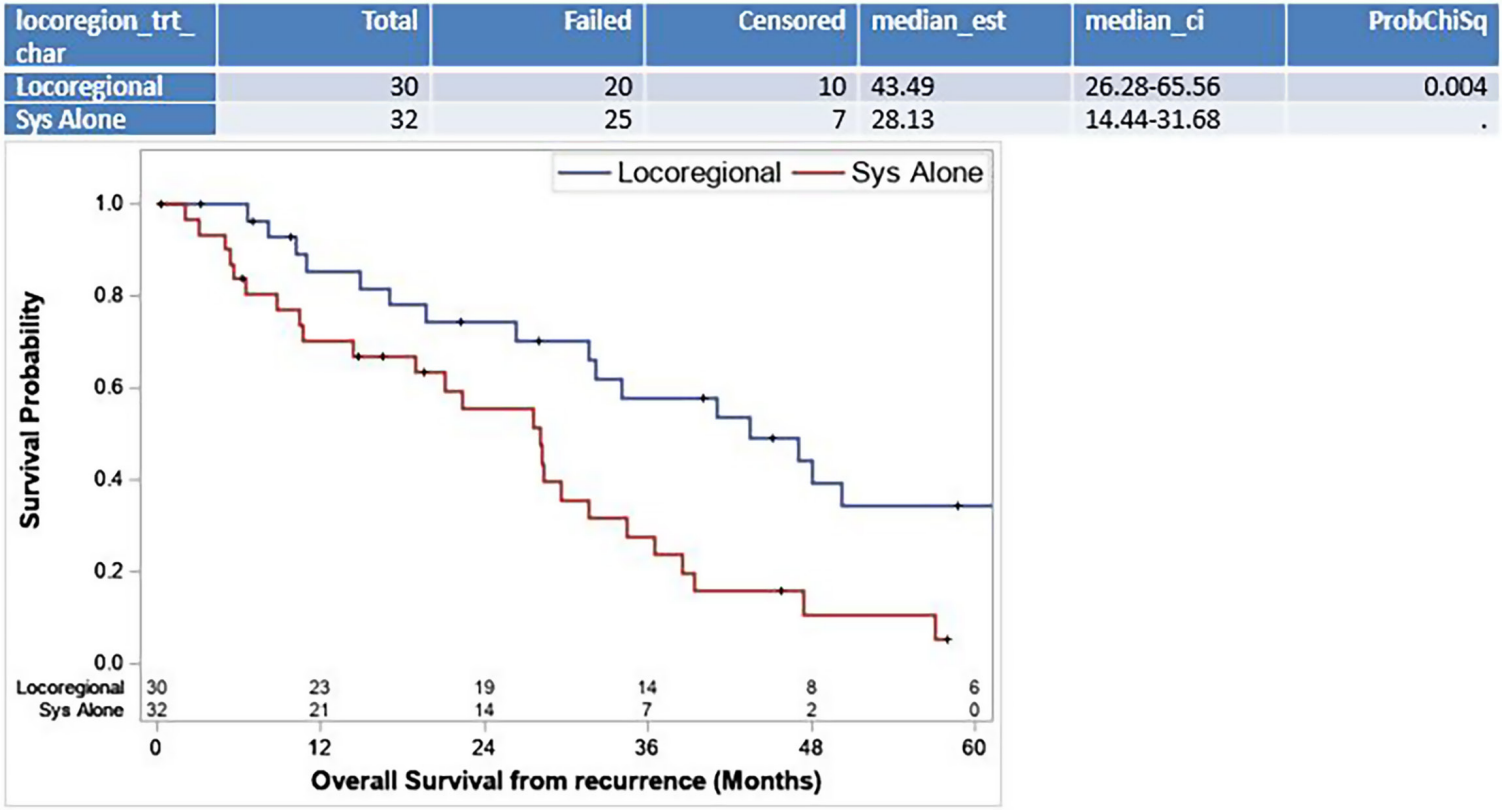
Overall survival of resected patients with liver recurrence treated with locoregional therapy versus none. Three patients were lost to follow‐up at the time of recurrence, therefore *n* = 62. SYS, systemic chemotherapy.

Of the 250 resected patients (Table S1), 24.8% (*n* = 62) received adjuvant chemotherapy and 6.4% (*n* = 16) received neoadjuvant therapy. One hundred sixty‐one patients (64.4%) recurred after resection. Multifocal liver recurrence was more common in patients who had a simultaneous liver and distant organ recurrence (DOR) (simultaneous recurrence = 43.8% vs. DOR alone 22.9%, vs. isolated liver recurrence = 24.6%; *p* = 0.049) (Table S2). Among these 161 recurrences, patients who had DOR alone had the longest disease‐free interval (18 vs. 11 vs. 8, *p* < 0.001). Of the 65 patients with liver‐only recurrence (LR), 30 (46.2%) were treated with liver‐directed therapies (Table 2). There were no differences in age, ECOG status, tumor size, margin status, or treatment with adjuvant therapy or systemic therapy at the time of recurrence in patients who received locoregional therapy for liver‐isolated recurrence compared to those who did not. Locoregional therapies included: resection in 11/30 (36.7%), HAIP in 9/30 (30.0%), transarterial chemoembolization (TACE) in 4/30 (13.3%), and ablation in 6/30 (20.0%). The median OS was significantly longer in patients treated with locoregional therapy versus SYS alone after isolated LR (43.5 months, 95% CI 26.3–65.6 vs. 28.1 months, 95% CI 14.4–31.7; *p* = 0.004) (Figure 2). On multivariable analysis of patients who recurred (*n* = 161), locoregional therapy after recurrence was significantly associated with improved OS after recurrence (hazard ratio [HR] 0.46; 95% CI 0.29–0.75; *p* = 0.002), while recurrence within 12 months of resection was significantly associated with decreased OS (HR 2.1; 95% CI 1.3–3.2; *p* < 0.001), while controlling for recurrence location (Table S3).

**TABLE 2:** Characteristics of patients with liver recurrence only after resection.

	Locoregional therapy (*n* = 30)	No locoregional therapy (*n* = 35)	*p*‐Value
Age (years), median (range)	63.5 (44.2–83.5)	66.1 (44.2–86.9)	0.465
Sex			
Female	15 (50)	20 (57.1)	0.623
Male	15 (50)	15 (42.9)	
Race			0.429
White	28/30 (93.3)	28/35 (84.8)	
Other	2/30 (6.7)	5/35 (15.2)	
Unknown	0	2	
Tumor size (cm), median (range)	5.8 (1.3–15.0)	6.5 (3.0–18.0)	0.087
Margin			
Negative	27 (90)	31 (88.6)	>0.95
Positive	3 (10)	4 (11.4)	
Pathologic lymph node status			
Negative	16 (53.3)	14 (40.0)	0.365
Positive	3 (10)	8 (22.9)	
Unknown	11 (36.7)	13 (37.1)	
Multifocal liver disease			
No	24 (80)	25 (71.4)	0.566
Yes	6 (20)	10 (28.6)	
Grade			
Well	1 (3.3)	0 (0)	0.312
Moderate	24 (80)	25 (71.4)	
Poor	5 (16.7)	10 (28.6)	
Adjuvant chemotherapy			
No	21 (70)	25 (71.4)	>0.95
Yes	9 (30)	10 (28.6)	
Age at recurrence (years), median (range)	65.8 (46.0–85.6)	67.5 (45.1–88.1)	0.655
Time of recurrence after resection (months), median (range)	14.2 (3.1–66.5)	9.5 (2.7–97.9)	0.063
<12 months	13 (43.3)	24 (68.6)	0.048
≥12 months	17 (56.7)	11 (31.4)	
Eastern Cooperative Oncology Group performance status at recurrence			
0	11/23 (47.8)	15/27 (55.6)	0.442
1	10/23 (43.5)	12/27 (44.4)	
2	2/23 (8.7)	0/27 (0)	
Unknown	7	8	
Systemic chemotherapy at recurrence			
No	12/30 (40.0)	5 (17.9)	0.086
Yes	18/30 (60.0)	23 (82.1)	
Unknown	0	7	

*Note*: Data are *n* (%) unless noted.

We next evaluated the outcomes of the 315 patients with liver‐limited, locally advanced unresectable, or liver‐limited multifocal disease without distant organ metastasis (Table S4). One hundred seventy‐two patients were treated with HAIP (54.6%), and 143 patients were treated with SYS (45.4%). SYS patients were more likely to be older (median 62.0 vs. 66.7 years; *p* = 0.001). There was no difference in multifocal disease or tumor grade. Sixty‐four percent (*n* = 91/143) of SYS patients received gemcitabine with either cisplatin or oxaliplatin as first‐line therapy. HAIP patients received FUDR alone (*n* = 51/172, 29.7%) or in combination with irinotecan (*n* = 37/172, 21.5%), gemcitabine and oxaliplatin (*n* = 46/172, 26.7%), or gemcitabine alone (*n* = 25/172, 14.5%).

In HAIP patients, 55 (32.0%) received SYS prior to HAIP. Of these patients, 21 (38.2%) progressed on systemic therapy prior to HAIP placement, 24 (43.6%) had stable disease, 3 (5.5%) responded to systemic therapy but remained unresectable, and 5 (9.1%) developed dose‐limiting toxicity. One patient had a HAIP placed but was unable to receive liver‐directed therapy; this patient was included in the HAIP cohort for analysis.

**FIGURE 3:**
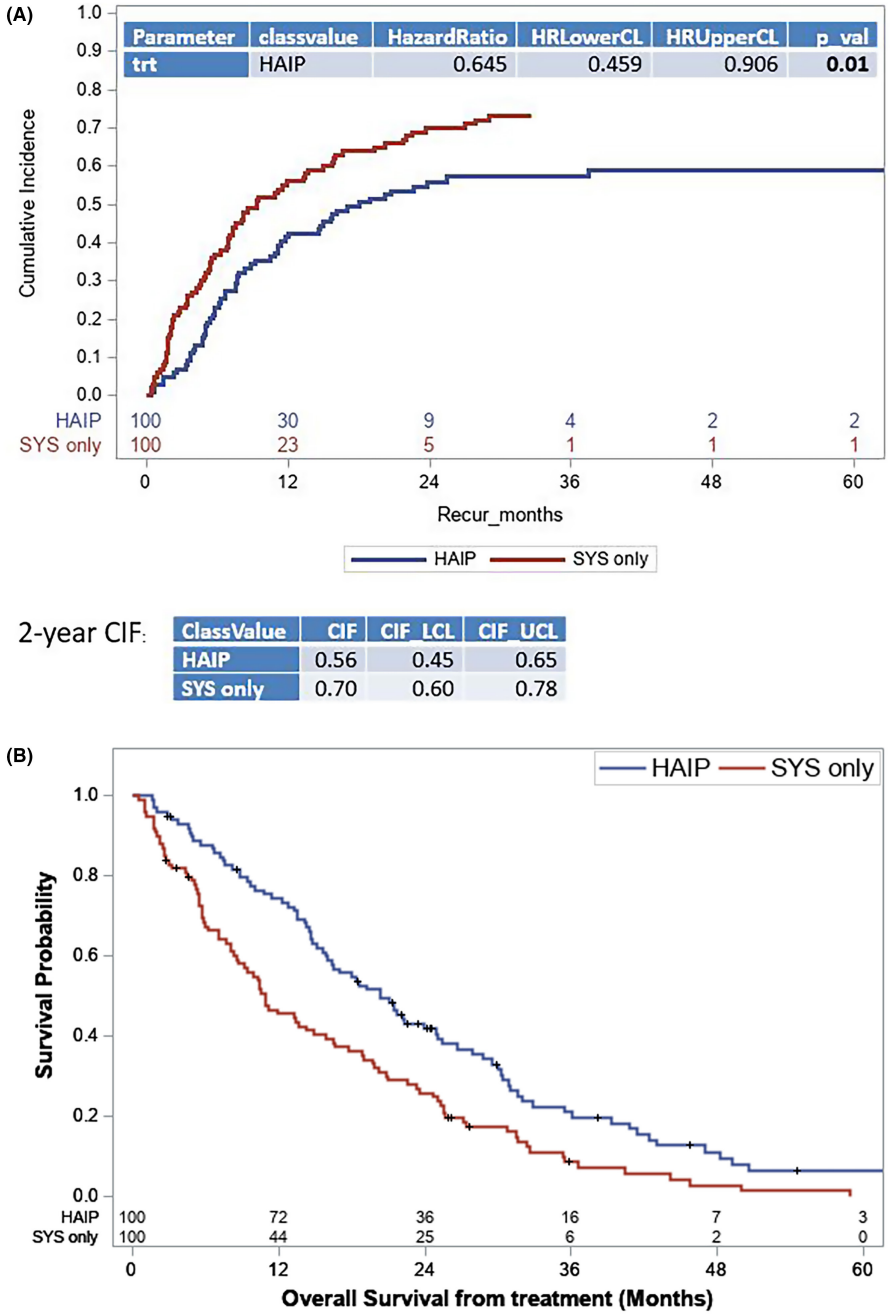
Propensity score matched analysis of (A) cumulative incidence of time to liver progression for patients and (B) overall survival of patients receiving hepatic artery infusion pump (HAIP; *n* = 100) versus systemic chemotherapy alone (SYS; *n* = 100). Progression is defined as liver progression alone or simultaneous liver and extrahepatic progression.

PSM was performed adjusting for age, sex, race, lymph node metastasis, multifocal liver disease, and tumor grade (Table 3 and Figure S2 **)**. After matching, HAIP was associated with improved hepatic PFS with HAIP (HR 0.65, 95% CI 0.46–0.91; *p* = 0.012) (Figure 3A) and longer OS (HR 0.59, 95% CI 0.43–0.80; *p* < 0.001) (HAIP [20.3 months, 95% CI 15.9–25.0] versus SYS [10.9 months, 95% CI 8.3–15.9]) (Figure 3B).

**TABLE 3:** Balance after propensity score matching of HAIP versus systemic chemotherapy in patients with intrahepatic locally advanced unresectable or multifocal liver disease only.

	Total (*n* = 200)	HAIP (*n* = 100)	SYS (*n* = 100)	*p*‐Value
Age (years), median (range)	64.7 (37.7–92.3)	64.1 (37.7–84.9)	65.3 (38.2–92.3)	0.503*
Sex				
Female	110 (55.0)	54 (54.0)	56 (56.0)	0.763**
Race				0.665**
White	176 (88.0)	89 (89.0)	87 (87.0)	
Other	24 (12)	11 (11)	13 (13)	
Suspicious lymph nodes				0.929***
None	75 (37.5)	38 (38.0)	37 (37.0)	
Regional	80 (40.0)	39 (39.0)	41 (41.0)	
Distant	17 (8.5)	7 (7.0)	10 (10.0)	
Regional + distant	28 (14.0)	16 (16.0)	12 (12.0)	
Multifocal liver disease	160 (80.0)	81 (81.0)	79 (79.0)	0.715**
Grade				0.703***
Well	5 (2.5)	2 (2.0)	3 (3.0)	
Moderate	115 (57.5)	57 (57.0)	58 (58.0)	
Poor	80 (40.0)	41 (41.0)	39 (39.0)	

*Note*: Data are *n* (%) unless noted.

Abbreviations: HAIP, hepatic arterial infusion pump; SYS, systemic chemotherapy only.

* Wilcoxon sign rank test.

** McNemar's test.

*** GEE model accounting for matching.

Finally, we analyzed the impact of liver progression and extrahepatic progression on OS in this propensity‐matched cohort. Progression in the liver, either alone or combined with extrahepatic recurrence/progression, was associated with a 10‐fold increased risk in mortality (compared to eightfold for extrahepatic disease alone) (Figure S3).

## 4 DISCUSSION

In this study, we demonstrate the importance of hepatic disease control to outcome in patients with IHC. For those eligible, curative‐intent resection provides the best hepatic disease control and survival. Although the median OS for curative‐intent resection in our series was encouraging at 48.1 months, intrahepatic recurrence is common, with a cumulative incidence of 41% at 2 years. For those patients with unresectable disease, HAIP‐delivered chemotherapy was associated with a 10.9‐month survival advantage compared to SYS alone. Moreover, recurrence or progression in the liver, either alone or simultaneously with extrahepatic recurrence or progression, was associated with a 10‐fold increased risk of mortality (compared to eightfold for extrahepatic disease alone). In a PSM cohort, patients treated with HAIP had a 35% lower risk of simultaneous intra‐ and extrahepatic progression and a 44% lower risk of isolated intrahepatic progression (compared to those treated with SYS alone). This was accompanied by a 9.4‐month survival difference in favor of HAIP between the two cohorts. Lastly, in resected patients with isolated LR, treatment with liver‐directed therapy was associated with a 15.4‐month improvement in median survival. Taken together, these data demonstrate both the adverse impact of intrahepatic recurrence/progression on survival, as well as the ability of HAIP to provide better hepatic disease control and improved survival compared to treatment with SYS alone.

In a systematic review and meta‐analysis by Choi et al. the authors identified four series describing early intrahepatic recurrence (defined as either 6, 12, or 24 months after resection) and noted a 49.6% 24‐month intrahepatic recurrence rate, similar to our finding of 41%.^18^ Unfortunately, the majority of patients will expereince liver recurrence, with most reports describing >50% isolated liver or simultaneous liver and extrahepatic recurrence.^9^, ^18^, ^19^ In the same report, the authors pooled prognostic factors and found that locoregional therapy in the form of adjuvant chemoradiation was the only factor associated with a decreased risk of intrahepatic recurrence (HR 0.68, 95% CI 0.49–0.93, *I* ^2^ = 0%). Similarly, in a retrospective series by Ercolani et al., the authors describe the treatment of patients with recurrent IHC and note that after 1999, 81.5% of patients received treatment for recurrence (of which 55.1% received locoregional therapy) and demonstrated a 26‐month median survival difference between those who did not receive treatment and those who did (20 vs. 46 months, respectively).^20^ In this same series, 84.6% of recurrences involved the liver, with 64.1% isolated intrahepatic and 20.5% simultaneous intra‐ and extrahepatic recurrence.

Yamashita et al. observed a higher risk of liver failure at the time of death in patients treated with chemotherapy compared to patients treated with radiation or resection.^21^ Additionally, local therapy was the sole predictor of death without liver failure on multivariable analysis.^21^ Consistent with prior studies, rates of intrahepatic progression and recurrence are significant; however, locoregional therapy (either in the form of HAIP or otherwise) is associated with both decreased hepatic disease recurrence/progression and survival. The benefit of local therapy may also extend to hepatocellular carcinoma (HCC). Ablation may convey some benefit for tumors <3 cm and multifocal disease; however, there is an increased risk of local recurrence compared to resection for larger tumors.^22^ However, almost two decades ago, TACE was demonstrated to confer a survival benefit compared to supportive treatment for patients with “intermediate stage” tumors (Barcelona Clinic Liver Cancer [BCLC] B).^23^, ^24^ More recently, a Phase III trial of HAI with fluorouracil, leucovorin, and oxaliplatin (FOLFOX) demonstrated a 7‐month survival benefit (23.1 vs. 16.1 months) compared to TACE for advanced, unresectable HCC.^25^

Although resection provides the best potential for a long‐term cure, disease‐specific factors, such as lymph node metastases and tumor multifocality (either satellite lesions or intrahepatic metastases), can significantly impact post‐resection survival.^26^, ^27^, ^28^ We recently demonstrated that for patients with lymph node metastases, there was no difference in survival between resection or HAIP (19.7 vs. 18.1 months, respectively).^29^ Similarly, using a multi‐institutional cohort of patients from 12 centers who underwent resection of multifocal IHC, there was no difference in survival compared to HAIP (18.9 vs. 20.3 months, respectively). Unfortunately, due to the rarity of IHC, available literature on other locoregional therapies is heterogeneous and of insufficient quality to generate high‐level recommendations. However, in a systematic review and pooled analysis by Edeline et al. the authors report that the pooled mean OS for intra‐arterial therapy, including selective internal radiation therapy, TACE, and HAIP, was 25.2 months for first‐line treatment with concomitant systemic chemotherapy.^30^ Thus, while resection remains the optimal treatment, liver‐directed chemotherapy may be as efficacious as resection at controlling liver disease in the case of adverse disease‐specific factors.

There are several limitations to this study, including its retrospective and single‐institution nature. Although the design employed was designed to limit selection bias, this can only be truly controlled by prospective randomization, and there may be other factors not in the cohort matching that might have influenced clinicians not to go with HAIP. The median OS of 10.9 months for SYS treated patients is lower than the landmark analysis of the ABC‐01, ‐02, and ‐03 trials which demonstrated a 15.4‐month median OS for liver‐limited disease.^12^ However, clinical trials result in a highly selected cohort of patients and similarly, in the Phase II trial of HAI‐FUDR for unresectable IHC conducted at our institution, the median OS was 25.0 months.^11^ Additionally, 31.3% of patients in the HAIP cohort received SYS prior to initiation of HAIP, which may have selected for patients with stable disease on systemic therapy. However, 32 were included in the PSM survival analysis and 11 were started on HAIP in the context of progression of disease, suggesting favorable disease biology did not artificially inflate survival data. Finally, although these data suggest an association of liver progression or recurrence with OS, the exact cause of death was not determined. Liver progression can lead to death in a number of ways, including hepatic failure, and uncontrolled cholangitis/sepsis, and while one or more of these mechanisms were likely involved, the precise mechanism cannot be ascertained in a retrospective analysis.

In conclusion, these data demonstrate the adverse impact of loss of liver disease control and the ability of liver‐directed chemotherapy to prevent intrahepatic recurrence/progression. These data demonstrate that liver‐specific treatment with locoregional therapy is an important consideration in addition to treatment with cytotoxic regimens in patients with IHC. Previous Phase II clinical trials have demonstrated the safety and potential efficacy of HAI FUDR in combination with gemcitabine and oxaliplatin for unresectable IHC.^31^ Our institution has an ongoing prospective trial that will compare this combination to gemcitabine and oxaliplatin alone (NCT04891289) to both determine the survival benefit of liver‐directed chemotherapy and better define factors associated with IHC pathogenesis and outcome.

## AUTHOR CONTRIBUTIONS

**Kevin C. Soares:** Conceptualization (equal); data curation (equal); formal analysis (equal); funding acquisition (equal); investigation (equal); methodology (equal); project administration (equal); resources (equal); software (equal); supervision (equal); validation (equal); visualization (equal); writing – original draft (equal); writing – review and editing (equal). **Joshua Samuel Jolissaint:** Conceptualization (equal); data curation (equal); formal analysis (equal); funding acquisition (equal); investigation (equal); methodology (equal); project administration (equal); resources (equal); software (equal); supervision (equal); validation (equal); visualization (equal); writing – original draft (equal); writing – review and editing (equal). **Sarah McIntyre:** Conceptualization (supporting); data curation (supporting); formal analysis (supporting); funding acquisition (supporting); investigation (supporting); methodology (supporting); project administration (supporting); resources (supporting); software (supporting); supervision (supporting); validation (supporting); visualization (supporting); writing – original draft (supporting); writing – review and editing (supporting). **Kenneth P. Seier:** Conceptualization (supporting); data curation (supporting); formal analysis (supporting); investigation (supporting); methodology (supporting); project administration (supporting); resources (supporting); software (supporting); supervision (supporting); validation (supporting); visualization (supporting); writing – original draft (supporting); writing – review and editing (supporting). **Mithat Gonen:** Conceptualization (equal); data curation (equal); formal analysis (equal); investigation (equal); methodology (equal); project administration (equal); resources (equal); software (equal); supervision (equal); validation (equal); visualization (equal); writing – original draft (equal); writing – review and editing (equal). **Carlie Sigel:** Conceptualization (supporting); investigation (supporting); methodology (supporting); supervision (supporting); validation (supporting); visualization (supporting); writing – original draft (supporting); writing – review and editing (supporting). **Naaz Nasar:** Data curation (supporting); writing – review and editing (supporting). **Andrea Cercek:** Conceptualization (supporting); investigation (supporting); methodology (supporting); supervision (supporting); validation (supporting); visualization (supporting); writing – original draft (supporting); writing – review and editing (supporting). **James J Harding:** Conceptualization (supporting); investigation (supporting); methodology (supporting); supervision (supporting); validation (supporting); visualization (supporting); writing – original draft (supporting); writing – review and editing (supporting). **Nancy E Kemeny:** Conceptualization (supporting); investigation (supporting); methodology (supporting); supervision (supporting); validation (supporting); visualization (supporting); writing – original draft (supporting); writing – review and editing (supporting). **Louise C Connell:** Conceptualization (supporting); investigation (supporting); methodology (supporting); supervision (supporting); validation (supporting); visualization (supporting); writing – original draft (supporting); writing – review and editing (supporting). **Bas Groot Koerkamp:** Conceptualization (supporting); investigation (supporting); methodology (supporting); supervision (supporting); validation (supporting); visualization (supporting); writing – original draft (supporting); writing – review and editing (supporting). **Vinod P. Balachandran:** Conceptualization (supporting); investigation (supporting); methodology (supporting); supervision (supporting); validation (supporting); visualization (supporting); writing – original draft (supporting); writing – review and editing (supporting). **Michael I D'Angelica:** Conceptualization (supporting); investigation (supporting); methodology (supporting); supervision (supporting); validation (supporting); visualization (supporting); writing – original draft (supporting); writing – review and editing (supporting). **Jeffrey A. Drebin:** Conceptualization (supporting); investigation (supporting); methodology (supporting); supervision (supporting); validation (supporting); visualization (supporting); writing – original draft (supporting); writing – review and editing (supporting). **T. Peter Kingham:** Conceptualization (supporting); investigation (supporting); methodology (supporting); supervision (supporting); validation (supporting); visualization (supporting); writing – original draft (supporting); writing – review and editing (supporting). **Alice C. Wei:** Conceptualization (supporting); investigation (supporting); methodology (supporting); supervision (supporting); validation (supporting); visualization (supporting); writing – original draft (supporting); writing – review and editing (supporting). **William Jarnagin:** Conceptualization (lead); data curation (equal); formal analysis (equal); funding acquisition (equal); investigation (equal); methodology (equal); project administration (lead); resources (lead); software (equal); supervision (lead); validation (equal); visualization (equal); writing – original draft (equal); writing – review and editing (equal).

## FUNDING INFORMATION

This work was supported in part by the Marie‐Josée and Henry R. Kravis Center for Molecular Oncology, the National Cancer Institute (P30‐CA008748; U01CA238444 to W.J.); and the National Center for Advancing Translational Sciences/Weill Cornell Medical College Clinical Translational Science Center (UL1‐TR002318444).

## CONFLICT OF INTEREST STATEMENT

ACW has received consulting fees from Biosapien and Histosonics. JJH has received research support from Bristol Myers Squibb and consulting fees from Bristol Myers Squibb, Merck, Eli Lilly, Eisai, Exelexis, Imvax, QED, and CytomX. NEK has received research funding from Amgen. VPB has received research funding from Bristol Myers Squibb and Genentech. TPK received one‐time compensation from Olympus.

## ETHICAL APPROVAL

Approval from the Institutional Review Board at Memorial Sloan Kettering Cancer Center (MSKCC) was obtained prior to data collection (protocol #16‐698).

## PATIENT CONSENT STATEMENT

N/A

## PERMISSION TO PRODUCE MATERIALS FROM OTHER SOURCES

N/A

## CLINICAL TRIAL REGISTRATION

N/A

## Supporting information


Figure S1:

## Data Availability

Data are available from the corresponding author upon request.
