# Supplementary material for: Hepatic disease control in patients with intrahepatic cholangiocarcinoma correlates with overall survival
Source: Cancer Med. 2023 Apr 16;12(11):12272–84. doi: 10.1002/cam4.5925 (PMC10278501; doi:10.1002/cam4.5925)
Supplement: Supplementary file 1 — Figure S1: [file CAM4-12-12272-s001.docx]

**Soares et al. Supplement**

**
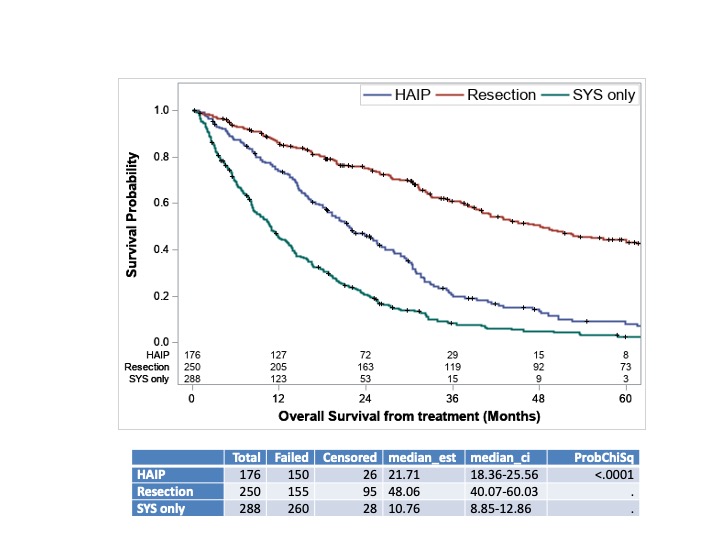
**

**A**

**B**

**
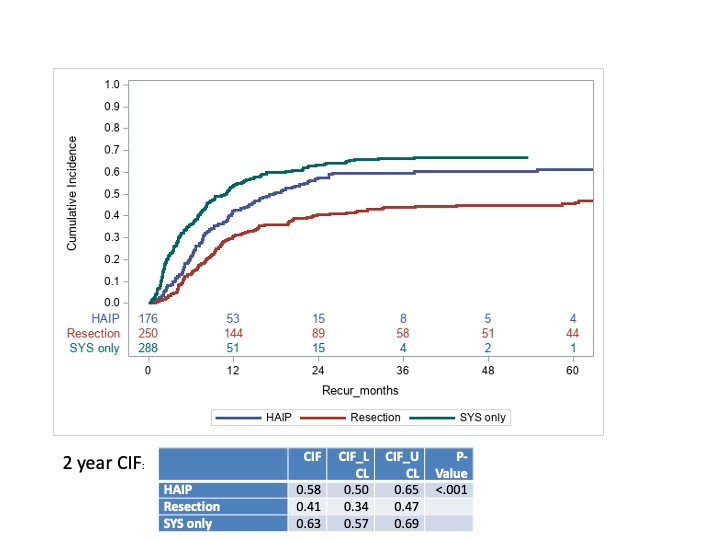
**

**Supplementary Figure 1**: **(A)** Overall survival according to treatment modality (upfront resection, hepatic artery infusion pump [HAIP], or systemic chemotherapy alone [SYS]) (n=714). **(B)** Cumulative incidence curve of time to progression or recurrence in the liver according to treatment modality (upfront resection, hepatic artery infusion pump [HAIP], or systemic chemotherapy alone [SYS]) (n=714).

**A**

**
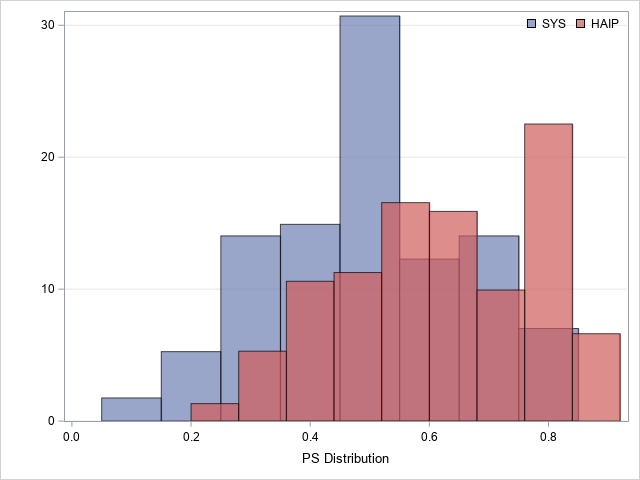
**

**B**

**
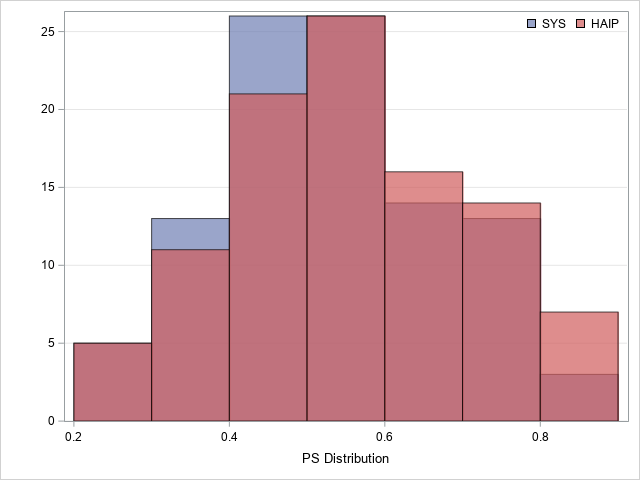
**

**Supplementary Figure 2:** Propensity score distribution pre (A) and post (B) matching.

**
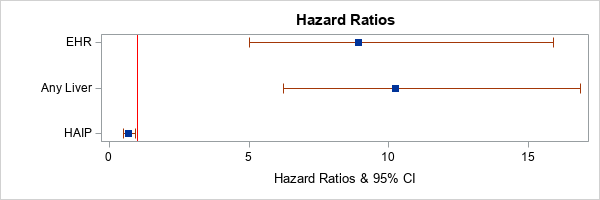

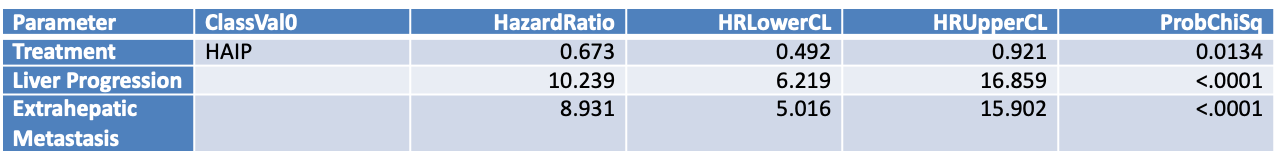
**

**Supplementary Figure 3:** Overall survival for propensity matched data with time dependent covariates for liver progression and development of extrahepatic metastasis (EHR).

**Supplementary Table 1**. Characteristics of resected patients (n=250)

| **Variable** | **n (%)** |
| --- | --- |
| Major hepatectomy |  |
| Extended right hepatectomy | 52 (20.8) |
| Right hepatectomy | 32 (12.8) |
| Extended left hepatectomy | 16 (6.4) |
| Left hepatectomy | 63 (25.2) |
| Central hepatectomy | 6 (2.4) |
| Minor hepatectomy |  |
| Posterior sectorectomy | 8 (3.2) |
| Left lateral sectorectomy | 11 (4.4) |
| Segmental resection | 61 (24.4) |
| Extrahepatic bile duct resection | 58 (23.2) |
| Vascular resection | 27 (10.8) |
| Caudate lobectomy | 42 (16.8) |
| Lymphadenectomy | 147 (58.8) |
| HAIP placement |  |
| Neoadjuvant | 14 (5.6) |
| Adjuvant | 8 (3.2) |
| Recurrence | 11 (4.4) |
| Systemic chemotherapy |  |
| Neoadjuvant | 16 (6.4) |
| Adjuvant | 62 (24.8) |
| R0 resection | 229 (91.6) |
| Lymph nodes removed, median (range)^a,b^ | 2 (1-16) |

^a^For those patients who had a lymphadenectomy performed

^b^11 patients lymphadenectomy was performed during neoadjuvant HAIP placement

Data are n (%) unless noted. HAIP = hepatic arterial infusion pump

**Supplementary Table 2:** Characteristics of recurred patients after curative resection

| **Variable** | **Total (n=161)** | **EHR (n=48)** | **LR (n=65)** | **Sim (n=48)** | **p-value** |
| --- | --- | --- | --- | --- | --- |
| Age at initial resection (years), median (range) | 66.0 (28.7-87.4) | 66.4 (40.3-83.1) | 65.7 (44.2-86.9) | 66.5 (28.7-87.4) | 0.847 |
| Sex, female | 89 (55.3) | 26 (54.2) | 35 (53.8) | 28 (58.3) | 0.920 |
| Race |  |  |  |  | 0.345 |
| Other | 25 (15.9) | 8 (17.4) | 7 (11.1) | 10 (20.8) |  |
| White | 132 (84.1) | 38 (82.6) | 56 (88.9) | 38 (79.2) |  |
| Unknown | 4 | 2 | 2 | 0 |  |
| Original tumor size (cm), median (range) | 6.2 (1.0-20.0) | 5.7 (2.5-20.0) | 6.0 (1.3-18.0) | 7.3 (1.0-18.5) | 0.150 |
| Negative margin at resection | 148 (91.9) | 46 (95.8) | 58 (89.2) | 44 (91.7) | 0.510 |
| Pathologic lymph node status |  |  |  |  | 0.034 |
| Negative | 70 (43.5) | 27 (56.3) | 30 (46.2) | 13 (27.1) |  |
| Positive | 36 (22.4) | 10 (20.8) | 11 (16.9) | 15 (31.3) |  |
| pXn | 55 (34.2) | 11 (22.9) | 24 (36.9) | 20 (41.7) |  |
| Multifocal liver disease | 48 (29.8) | 11 (22.9) | 16 (24.6) | 21 (43.8) | 0.049 |
| Grade |  |  |  |  | 0.006 |
| Well differentiated | 1 (0.6) | 0 (0) | 1 (1.5) | 0 (0) |  |
| Moderately differentiated | 102(64.6) | 31 (67.4) | 49 (75.4) | 22 (46.8) |  |
| Poorly differentiated | 55(34.8) | 15 (32.6) | 15 (23.1) | 25 (53.2) |  |
| Unknown | 3 | 2 | 0 | 1 |  |
| Adjuvant chemotherapy | 61 (37.9) | 19 (39.6) | 19 (29.2) | 23 (47.9) | 0.131 |
| Age at recurrence (years), median (range) | 67.9 (30.1-88.1) | 68.0 (42.3-85.1) | 67.1 (45.1-88.1) | 68.3 (30.1-87.9) | 0.702 |
| Disease-free interval, months, median (range) | 10.6 (1.0-126) | 18.3 (2.6-91.9) | 11.1 (2.7-97.9) | 7.9 (1.0-126) | <.001 |
| Less than 1 year DFI | 87 (54.0) | 12 (25) | 37 (56.9) | 38 (79.2) | <.001 |
| Locoregional therapy | 42 (26.1) | 3 (6.3) | 30 (46.2) | 9 (18.8) | <.001 |
| ECOG performance status at recurrence |  |  |  |  | 0.224 |
| 0 | 29 (45.3) | 1 (25.0) | 26 (52.0) | 2/ (20.0) |  |
| 1 | 33 (51.6) | 3 (75.0) | 22 (44.0) | 8 (80.0) |  |
| 2 | 24 (3.1) | 0 (0) | 2 (4.0) | 0 (0) |  |
| Unknown | 97 | 44 | 15 | 38 |  |

Data are n (%) unless noted. DFI = disease-free interval; EHR = extrahepatic recurrence; ECOG = Eastern Cooperative Oncology Group; LR = liver recurrence; Sim = simultaneous liver and extrahepatic recurrence

**Supplementary Table 3**: Univariate and multivariable analysis of overall survival after recurrence

**Univariable Analysis**

| **Parameter** | **Class** | **Reference** | **Hazard Ratio** | **95% CI** | **p-value** |
| --- | --- | --- | --- | --- | --- |
| Recurrence site status | LR | EHR | 0.721 | (0.461-1.126) | 0.150 |
|  | Sim | EHR | 1.152 | (0.730-1.818) | 0.542 |
| Recurrence |  |  | 0.986 | (0.973-0.999) | 0.041 |
| 1 year RFS (months) | <12 | ≥12 | 1.840 | (1.278-2.649) | 0.001 |
| Locoregional treatment | Yes | No | 0.419 | (0.269-0.654) | <0.001 |

**Multivariable Analysis**

| **Parameter** | **Class** | **Hazard Ratio** | **95% CI** | **p-value** |
| --- | --- | --- | --- | --- |
| Recurrence site status | LR | 0.699 | (0.410-1.193) | 0.189 |
|  | Sim | 0.904 | (0.536-1.525) | 0.705 |
| 1 year RFS (months) | <12 | 2.065 | (1.349-3.162) | <0.001 |
| Locoregional treatment | Yes | 0.464 | (0.288-0.749) | 0.002 |

EHR = extrahepatic recurrence; LR = liver recurrence; RFS = recurrence-free survival; Sim = simultaneous liver and extrahepatic recurrence; CI = confidence interval

**Supplementary Table 4.** Clinicopathologic characteristics of patients with intrahepatic locally advanced unresectable or multifocal liver disease only stratified by treatment modality (n=315)

| **Variable** | **HAIP (n=172 )** | **SYS (n=143)** | **p-value** |
| --- | --- | --- | --- |
| Age (years), median (range) | 62.0 (30.1-85.7) | 66.7 (32.2-92.3) | 0.001 |
| Sex, female | 104 (60.5) | 75 (52.4) | 0.171 |
| Race |  |  | 0.084 |
| White | 151 (90.4) | 114 (83.2) |  |
| Other | 16 (9.6) | 23 (16.8) |  |
| Multifocal liver disease | 126 (73.3) | 107 (74.8) | 0.797 |
| Grade |  |  |  |
| Well differentiated | 6 (3.9) | 3 (2.5) | 0.727 |
| Moderately differentiated | 86 (55.5) | 71 (59.7) |  |
| Poorly differentiated | 63 (40.6) | 45 (37.8) |  |
| Unknown | 17 | 24 |  |
| Suspicious lymph nodes |  |  |  |
| None | 70 (40.7) | 55 (38.5) | <0.001 |
| Regional | 46 (26.7) | 57 (39.9) |  |
| Distant | 9 (5.2) | 15 (10.5) |  |
| Both | 47 (27.3) | 16 (11.2) |  |
| First line systemic chemotherapy |  |  |  |
| Gemcitabine alone | 1 (0.6) | 15 (10.5) |  |
| Gemcitabine/cisplatin | 27 (15.7) | 65 (45.5) |  |
| Gemcitabine/oxaliplatin | 9 (5.2) | 26 (18.2) |  |
| Gemcitabine/capecitabine | 2 (1.2) | 3 (2.1) |  |
| Gemcitabine/irinotecan | 0 (0) | 3 (2.1) |  |
| FOLFOX/XELOX | 2 (1.2) | 7 (4.9) |  |
| FOLFIRI/XELIRI | 1 (0.6) | 0 (0) |  |
| Flourouracil or capecitabine monotherapy | 0 (0) | 8 (5.6) |  |
| Other | 11 (6.4) | 15 (10.5) |  |
| ≥2 lines of chemotherapy | 15 (8.7) | 74 (51.7) |  |
| HAIP chemotherapy regimen |  | N/A |  |
| FUDR monotherapy | 51 (29.7) | N/A |  |
| FUDR + irinotecan | 37 (21.5) | N/A |  |
| FUDR + gemcitabine/oxaliplatin | 46 (26.7) | N/A |  |
| FUDR + gemcitabine | 25 (14.5) | N/A |  |
| FUDR + avastin | 9 (5.2) | N/A |  |
| FUDR + other agents | 3 (1.7) |  |  |

Data are n (%) unless noted; FUDR = floxuridine; HAIP = hepatic arterial infusion pump; SYS = systemic chemotherapy only
